# Tuning of hydrogel stiffness using a two‐component peptide system for mammalian cell culture

**DOI:** 10.1002/jbm.a.36568

**Published:** 2018-11-19

**Authors:** Alessandra Scelsi, Brigida Bochicchio, Andrew Smith, Victoria L. Workman, Luis A. Castillo Diaz, Alberto Saiani, Antonietta Pepe

**Affiliations:** ^1^ Laboratory of Bioinspired Materials, Department of Science University of Basilicata Potenza Italy; ^2^ PhD School of Science, University of Basilicata Potenza Italy; ^3^ School of Materials and Manchester Institute of Biotechnology, The University of Manchester Manchester United Kingdom; ^4^ Biotecnología Médica y Farmacéutica. Centro de Investigación y Asistencia en Tecnología y Diseño del Estado de Jalisco (CIATEJ) Guadalajara Mexico

**Keywords:** self‐assembling peptides, hydrogel, atomic force microscopy, elastin sequence, oscillatory rheology, cell culture

## Abstract

Self‐assembling peptide hydrogels (SAPHs) represent emerging cell cultures systems in several biomedical applications. The advantages of SAPHs are mainly ascribed to the absence of toxic chemical cross‐linkers, the presence of ECM‐like fibrillar structures and the possibility to produce hydrogels with a large range of different mechanical properties. We will present a two‐component peptide system with tuneable mechanical properties, consisting of a small pentapeptide (SFFSF‐NH_2_, SA5N) that acts as a gelator and a larger 21‐mer peptide (SFFSF‐GVPGVGVPGVG‐SFFSF, SA21) designed as a physical cross‐linker. The hydrogels formed by different mixtures of the two peptides are made up mainly of antiparallel β‐sheet nanofibers entangling in an intricate network. The effect of the addition of SA21 on the morphology of the hydrogels was investigated by atomic force microscopy and transmission electron microscopy and correlated to the mechanical properties of the hydrogel. Finally, the biocompatibility of the hydrogels using 2D cell cultures was tested. © 2018 The Authors. journal Of Biomedical Materials Research Part A Published By Wiley Periodicals, Inc. J Biomed Mater Res Part A: 107A: 535–544, 2019.

## 1 INTRODUCTION

In the last decade, hydrogels formed from self‐assembling peptides were recognized as useful scaffolds for cell culture and tissue regeneration.^1^, ^2^, ^3^ Self‐assembling peptide hydrogels (SAPHs) are advantageous as they are easy to produce, non‐toxic, biocompatible, biodegradable, often injectable and as such applicable as scaffolds for tissue engineering as well as for drug delivery systems.^4^, ^5^, ^6^ A number of successful studies have demonstrated the feasibility of using peptide‐based hydrogels as scaffolds for a high number of cell types.^7^, ^8^, ^9^, ^10^ The main advantages of SAPHs over other types of hydrogels is the absence of harmful chemicals (e.g., toxic cross‐linkers) to obtain the hydrogels, high internal hydration and a fibrous peptide nano‐architecture, mimicking collagen fibers that offer a valid choice as extracellular matrix (ECM) mimics.^3^, ^11^, ^12^, ^13^, ^14^, ^15^ However, the molecular design of a self‐assembling peptide system to be developed as scaffold in cell cultures, has to consider that the ECM is a dynamic environment with particular mechanical properties and is constantly remodeled by various enzymes and cells present in the ECM responsible for its synthesis, organization, maintenance, and degradation.^12^, ^16^ Recently, several studies have focused on the impact of the mechanical properties of the matrix on the normal development of tissue during embryogenesis and growth, highlighting the influence of the stiffness of the substrates on cell behavior.^17^, ^18^ In this context, the possibility to control the mechanical properties of the matrix is of paramount importance, in order to realize hydrogels for different cells.

Several studies have shown peptide hydrogel systems with tuneable stiffness, where tuning is mainly achieved by varying peptide concentrations in the hydrogels.^19^, ^20^, ^21^ Some attempts to change hydrogel stiffness by varying amino acid composition in peptide amphiphilic systems has been explored, highlighting the key role of hydrophobic interactions in gel stiffness.^22^, ^23^ A similar effect on gel strength can be obtained by varying salt concentration, when charged amino acids are present in the peptide sequence.^24^, ^25^ More drastic strengthening of the hydrogel can be obtained by chemical crosslinking, like oxidation of cysteine residues belonging to the peptide sequence or by irradiation once non‐natural amino acids are introduced in the peptide.^26^, ^27^, ^28^

An alternative strategy to tune properties of the hydrogel is to use multicomponent systems, where the mixed self‐assembling peptides can differ with point amino acid substitution, or can be unrelated.^22^, ^29^, ^30^, ^31^, ^32^ Another example that improved peptide gel strength and systematically controlled the mechanical properties of peptide hydrogels, was based on mixing various quantities of self‐assembling ionic‐complementary peptide FEFEFKFK (FEKII8) and its double length counterpart FEFEFKFK‐GG‐FKFKFEFE (FEKII18).^20^ While the hydrogels obtained in water solutions show a G′ for FEKII8 of 80 ± 5 Pa, when FEKII18 is added to FEKII8 network an increase of G′ of 30 times is achieved. However, the reached stiffness, while appropriate for cells growing on soft tissues would not be appropriate for other applications in tissue engineering, such as cartilage or bone scaffolds.

We propose a two‐component peptide system designed to produce a wide range of stiffness values for the hydrogel by the appropriate combination of two designed self‐assembling peptides. The peptides are SFFSF‐NH_2_ (SA5N) and SFFS‐FGVPGVGVPGVG‐SFFSF (SA21). The proposed way to control mechanical properties of the hydrogels is by introducing physical crosslinks in the nanofibers structure by an elastin‐inspired three‐block peptide that contains at both ends sequences identical to the self‐assembling peptide.^33^ We evaluate how the addition of different amounts of the crosslinking peptide affects the mechanical properties of the hydrogels. The prepared hydrogels were characterized by various techniques including FTIR, AFM, TEM, and oscillatory shear rheology. Furthermore, the biocompatibility of the hydrogels using 2D cell cultures was investigated.

## 2 MATERIALS AND METHODS

### 2.1 Peptide synthesis

The peptides, SA5N and SA21 were synthesized by solid phase peptide synthesis (SPPS) on a Tribute automatic peptide synthesizer (Protein Technologies Inc.) with a standard 9‐fluorenylmethoxycarbonyl (Fmoc) protection peptide synthesis protocol. Fmoc‐α‐amino acids were purchased from Inbios (Pozzuoli, Italy), and coupling reagent *N*‐[(1*H*‐benzotriazol‐1‐yloxy)(dimethylamino) methylene]‐*N*‐methylmethaniminium hexafluorophosphate (HBTU) was acquired from Matrix Innovation (Quebec, Canada). Reagent used for cleavage from resin was an aqueous solution of 95% 2,2,2‐trifluoroacetic acid, (TFA, >99%). The peptides were purified by semi‐preparative reversed‐phase HPLC, using binary gradient H_2_O (0.1% TFA) and CH_3_CN (0.1 %TFA) as solvents. The purity of the peptides was assessed by ^1^H‐NMR spectroscopy and MS (ESI) mass spectrometry (Figs. S1–S6).

### 2.2 Hydrogel preparation

Hydrogels at a final concentration of 0.7%, 1% and 2% (w/v) were prepared by dissolving the weighed SA5N peptide powder in 10 μL of dimethyl sulfoxide (DMSO) and 200 μL of milliQ water. The solution was mildly agitated (5 min) at 100°C and then left to gel for 1 h. Hydrogels composed of a blend of SA5N and SA21 were prepared by adding SA21 dissolved in pure DMSO to the weighed amount of SA5N powder, followed by MilliQ water to a final concentration of 5% of DMSO. The mixture was mildly agitated (5 min) at 100°C and then left to gel for 1 h.

### 2.3 Fourier transform infrared spectroscopy

About 1–2% of lyophilized hydrogel was mixed with potassium bromide (KBr), ground to a fine powder and pressed to form a pellet. IR spectra were recorded on a Jasco FTIR‐460 spectrometer. Each spectrum is the result of signal‐averaging of 256 scans at a resolution of 2 cm^−1^. All spectra are absorbance spectra obtained after background subtraction. Smoothing of spectra was carried out with a step of 11 or 13 data points, using the Savitzky–Golay function. Second derivatives of the spectra were obtained using a step of 13 datapoints to identify discrete absorption bands that make up the complex amide profiles. Quantitative analysis of the individual component bands of amide I was achieved by using the peak fitting module implemented in the Origins® Software (Microcalc Inc.).

### 2.4 Oscillatory shear rheology

Rheological studies were performed on TA instrument discovery HR‐2 rheometer equipped with a Peltier device to control the temperature. Parallel plate geometry with 20 mm diameter with a 0.250 mm gap was used. To minimize the evaporation a solvent trap was applied. Initially, strain amplitude sweeps (*γ* = 0.04–10%) were performed at constant angular frequency (*ω* = 1 rad s^−1^) to identify the linear viscoelastic region (LVR). Subsequently, frequency sweeps (*ω* = 0.06–94.2 rad s^−1^) at constant strain amplitude (*γ* = 0.2%) were performed to determine the elastic (G′) and viscous (G″) moduli within the LVR. All measurements were performed at 25°C, and all measurements were repeated at least three times to ensure reproducibility.

### 2.5 Atomic force microscopy

Samples were prepared after 24 h of gelling by diluting the hydrogels tenfold. A 10 μL aliquot of the sample was then deposited onto a silicon wafer and air‐dried prior to imaging. Samples were stored in a Petri dish until observed by the scanning force microscope (Park Autoprobe XE‐120) at room temperature. Data acquisition was carried out in intermittent contact mode at scan rates between 0.2 Hz and 1.5 Hz, using rectangular Si cantilevers (TAP300Al‐G, Budget Sensors) having a radius of curvature less than 10 nm and with the nominal resonance frequency and force constant of 300 kHz and 40 N m^−1^, respectively.

### 2.6 Transmission electron microscopy

Samples were prepared after 24 h of gelling by diluting the hydrogel tenfold. The solutions were vortexed until they were fully homogeneous. A total of 10 μL of sample was deposited on a carbon coated copper grid (400 mesh, Agar Scientific) for 1 min. The loaded grids were negatively stained with freshly prepared and filtered 2% (w/v) uranyl acetate for 2 min and then washed with double‐distilled water twice, blotting at each stage using Whatman filter paper. After air‐drying, grids were observed by transmission electron microscopy (Fei Tecnai G2 20 Twin) operating at 100 kV. Fiber width and morphology analysis was performed by ImageJ.

### 2.7 Cell culture

Mouse NIH 3T3 fibroblasts were cultured in Dulbecco's modified Eagle's medium (DMEM) containing 10% foetal bovine serum (FBS) and 1% penicillin and streptomycin. Cells were maintained in a humidified incubator at 37°C with 5% CO_2_. All cell culture reagents were purchased from Invitrogen, U.K. Cell morphology was observed with a Leica light microscope.

### 2.8 2D cell culture hydrogels

While the gels were cooling they were pipetted into 12‐well cell culture inserts (ThinCert™ Greiner Bio‐One) (0.3 mL gel insert^−1^). Once cooled (5 min) the media (DMEM) was added on above (0.5 mL) and below (1.5 mL). Further media changes (3×) within 1 h were carried out at 37°C to obtain pH 7. Subsequently, 500 μL of cell suspension (3 × 10^4^ cells) was transferred to the surface of each gel. The hydrogels with cells were incubated at 37°C and confocal microscope images were collected at 5 h, 24 h, and 48 h.

### 2.9 Cell viability assay

Cell viability was qualitatively tested using a live/dead assay (Invitrogen) following instruction of the manufacturer. PBS containing 6.4 × 10^−6^ *M* ethidium homodimer‐1 (EthD‐1) and 2 × 10^−6^ *M* calcein AM was prepared. The live/dead assay solution was pipetted on top of samples, which were incubated for 30 min at 37°C. Thereafter, the staining solution was removed. Imaging of samples was obtained by Leica TCS SP5 confocal microscope at 2 h, 24 h, and 48 h.

### 2.10 Cell proliferation assay

Quantitative cell proliferation was evaluated using Alamar blue (AB) assay, which measures the metabolic activity of cells through a redox reaction. The fluorescence derived from the reduction of the oxidized Alamar Blue is an indicator of aerobic respiration.^34^

Alamar blue solution (10%) was added on top of samples 3 h before the timepoint, incubated for 3 h at 37°C and then removed into a 96 well plate for analysis at the following timepoints; (3 h, 24 h, and 48 h). Samples were analyzed at 545 nm/590 nm (excitation/emission) using the BMG LABTECH plate reader. The number of viable cells is proportional to the level of fluorescence. Duplicates were used.

## 3 RESULTS

### 3.1 Peptide design

**Figure 1:**
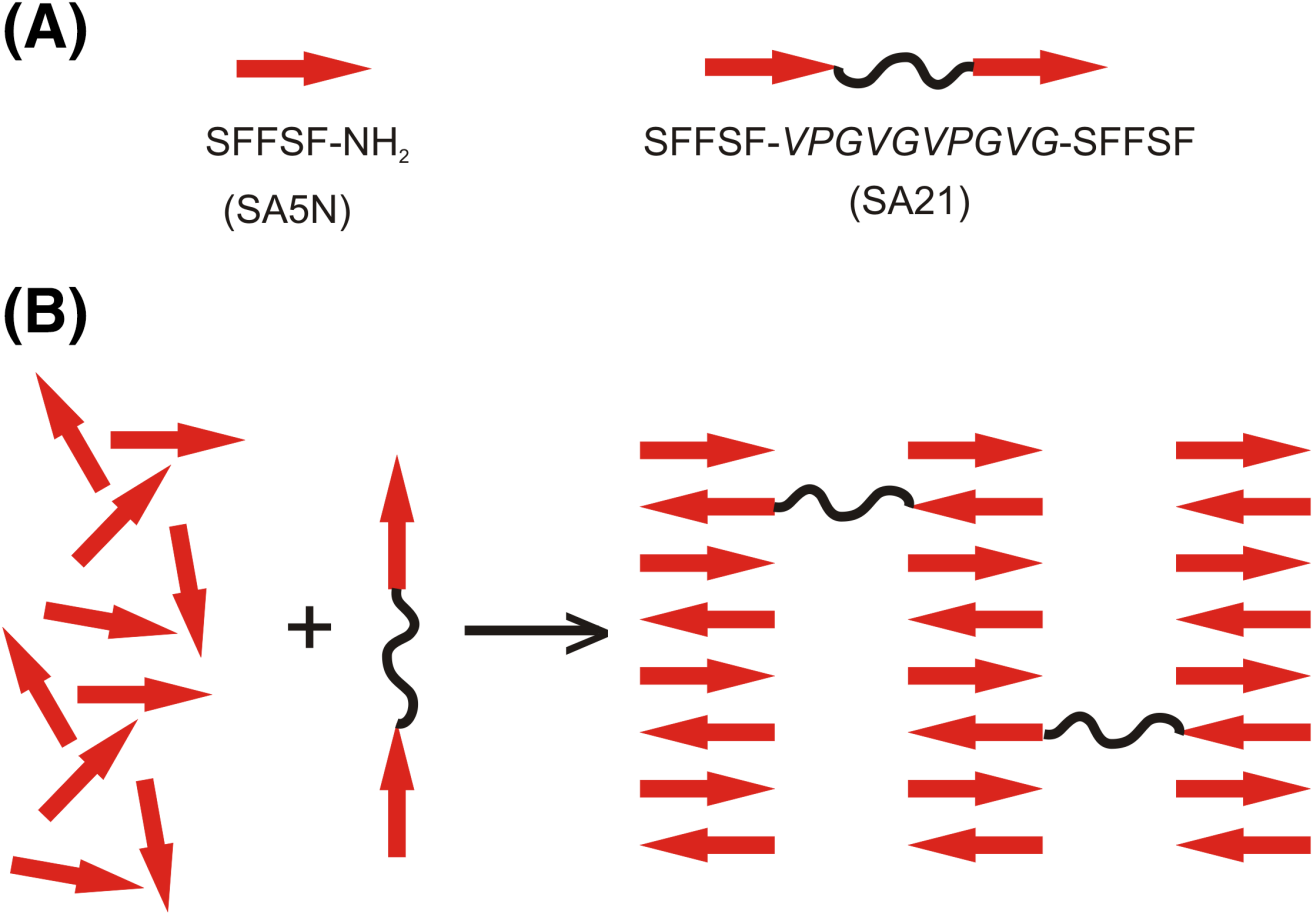
Schematic representation of the two‐component peptide system. (A) SA5N peptide and SA21 peptide; (B) proposed mechanism of two‐component hydrogel formation.

The aim of the present work is to design a two‐component peptide system that forms hydrogels with tuneable mechanical properties. The system is composed of a basic self‐assembling peptide (gelator) and a bridging peptide (physical cross‐linker) that combined in different ratios can modulate the stiffness of the hydrogels by introducing physical cross‐links between nanofibers (Fig. 1). The gelator peptide was based on the sequence SFFSF (SA5), corresponding to the 2–6 sequence fragment in the N‐terminal region of human serum amyloid A protein (hSAA1).^35^, ^36^ This protein and its truncated forms are responsible for secondary systemic amyloidosis observed in kidneys and liver during chronic inflammation.^37^, ^38^ SA5 peptide is able to form hydrogels in very harsh conditions (low or high pH), and thus is not suitable as a cell culture scaffold.^39^ Hence, to promote gelation of such type of peptides under physiological conditions, amidation of the C‐terminus was introduced in SA5 peptide sequence.^39^, ^40^ This modification rendered SFFSF‐NH_2_ peptide (SA5N) positively net charged at physiological conditions, avoiding precipitation. The introduction of the amide group shifts the isoelectric point from pH 6 to pH 14, which promotes the gelation at pH 7.^35^ The second component of the gelation system is SFFSF‐VPGVGVPGVG‐SFFSF (SA21) peptide, proposed as a physical cross‐linker. SA21 peptide was designed as an ABA three block system having two external β‐sheet forming sequences, corresponding to SA5, and a central flexible peptide sequence inspired by the consensus sequence –VGVPG– of elastin.^33^, ^41^

### 3.2 Hydrogel formation

**Figure 2:**
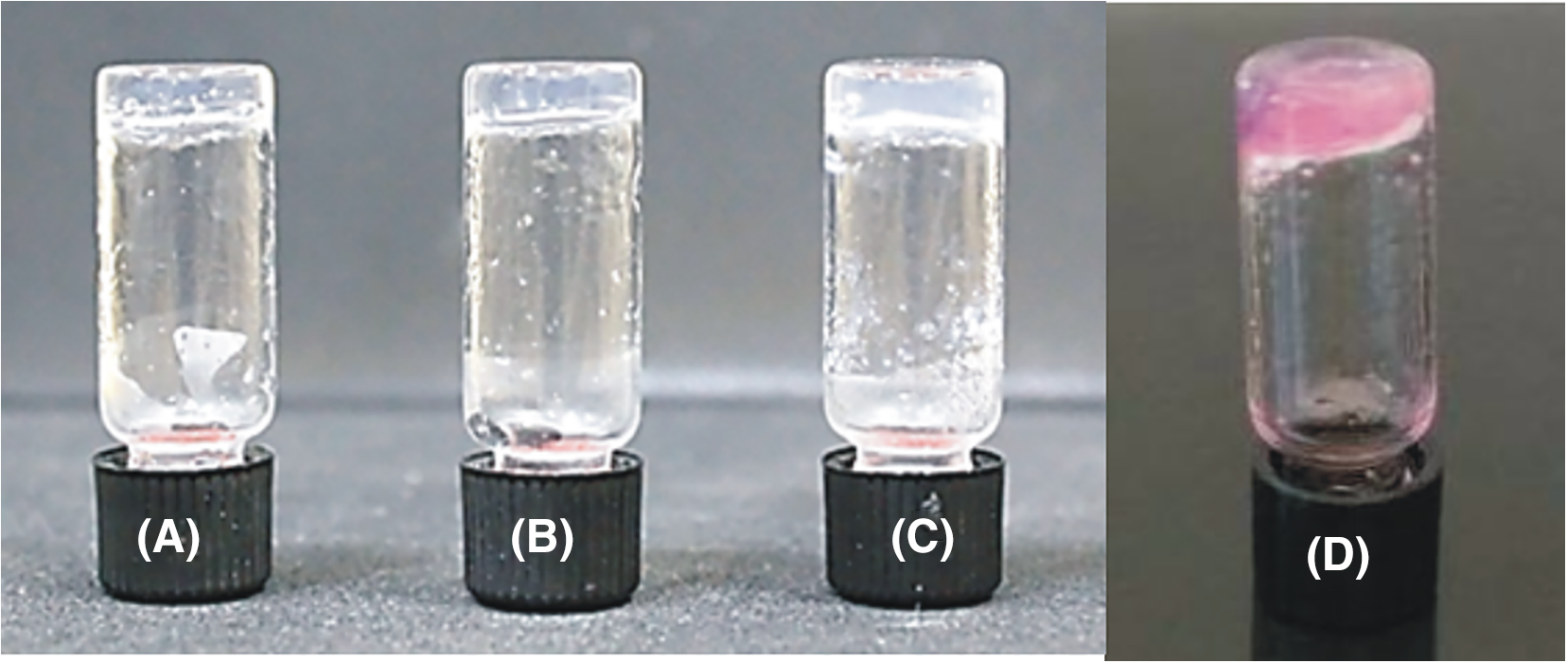
Self‐supporting hydrogel formation of SA5N at 0.7% (w/v) (A), 1.0% (w/v) (B); and 2.0% (w/v) (C); 1% at pH 7 with DMEM (D).

The SA5N peptide‐based hydrogel formation at different concentrations was visually monitored by inversion test. It is considered successful if the hydrogels are self‐supporting and do not flow upon inversion of the vial, as shown in Figure 2. Low SA5N peptide concentrations (0.7% w/v) led to weak gels, while higher peptide concentrations (1% and 2% w/v) resulted in stronger hydrogels. Hydrogels transparency decreases as peptide concentration increases.^42^ For both, SA5N and SA5N/SA21 peptide based hydrogels, cell culture media (DMEM) exchanges were carried out to obtain a physiological pH. In Table 1, a summary of the prepared hydrogels is presented.

**Table 1:** Summary of Peptide Hydrogels Composition

Hydrogel	SA5N (w/v)	SA21(w/v)
H1	0.7%	–
H2	1.0%	–
H3	2.0%	–
H4	1.0%	0.017%
H5	1.0%	0.17%
H6	2.0%	0.034%
H7	2.0%	0.34%

### 3.3 Fourier transform infrared spectroscopy

**Figure 3:**
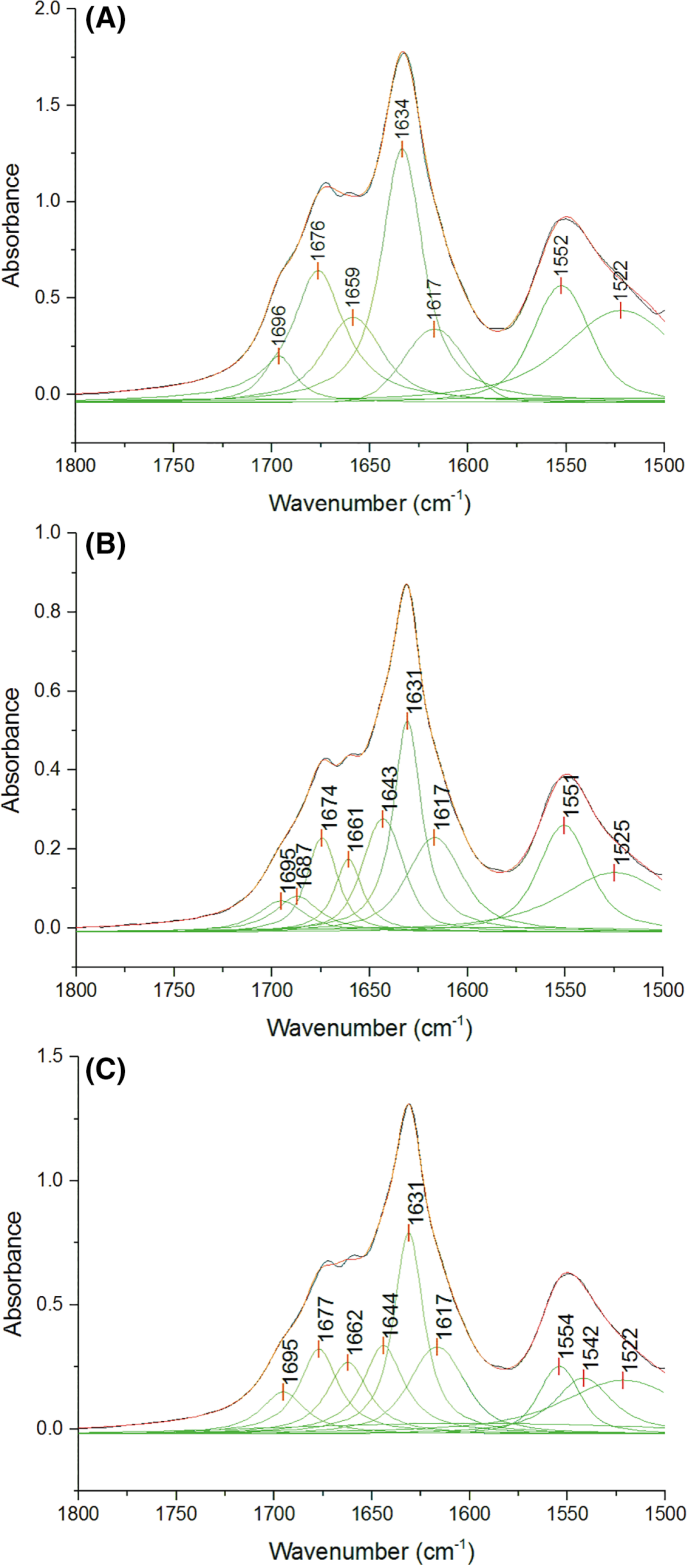
Decomposed FT‐IR spectra of H2 (A), H4 (B) and H5 (C) hydrogels, recorded as KBr pellets. The band fitting results of amide I and II regions are shown. Dashed line: experimental spectra. Solid red line: calculated spectra.

Insights into the molecular structure of the peptides forming the hydrogel network were gained by Fourier transform infrared (FT‐IR) spectroscopy. This technique is useful for determining the secondary structure of the peptide chain. In particular, Amide I and Amide II bands are very sensitive to the conformational state of the peptides. The decomposed amide I and II FTIR spectra of H2, H4, and H5 hydrogels are shown in Figure 3(A–C). Similar spectra are observed for the three samples, with a predominant band in the amide I region centered at 1631–1634 cm^−1^, assigned to β‐sheet structures. The concomitant presence of a high wavenumber band at 1695 and 1696 cm^−1^ suggests that the β‐sheet structure could be antiparallel. In all the samples, a low wavelength band at 1617 cm^−1^ is also present, assigned to a strongly H‐bonded amide group, usually observed in intermolecular aggregates and amyloid cross‐β structures.^43^ When SA21 is incorporated to the system, some differences in the spectra can be observed in the 1640–1665 cm^−1^ range assigned to unordered and loop structures. The results are compatible with the presence of a central elastin sequence that previous studies on elastin peptides have shown to adopt type II β‐turn together with unordered conformations.^44^, ^45^, ^46^, ^47^ In the Amide II region, where the absorption is due primarily to the bending vibration of the N–H bond, sensitive to H‐bonding, the two predominant bands are at ca. 1552 cm^−1^ and 1522 cm^−1^, assigned to β‐structures. The spectrum of H5 hydrogel shows an additional band at 1542 cm^−1^, assigned to unordered conformations, which might be due to the higher amount of SA21 peptide present in the hydrogel.

### 3.4 Shear oscillatory rheology

**Figure 4:**
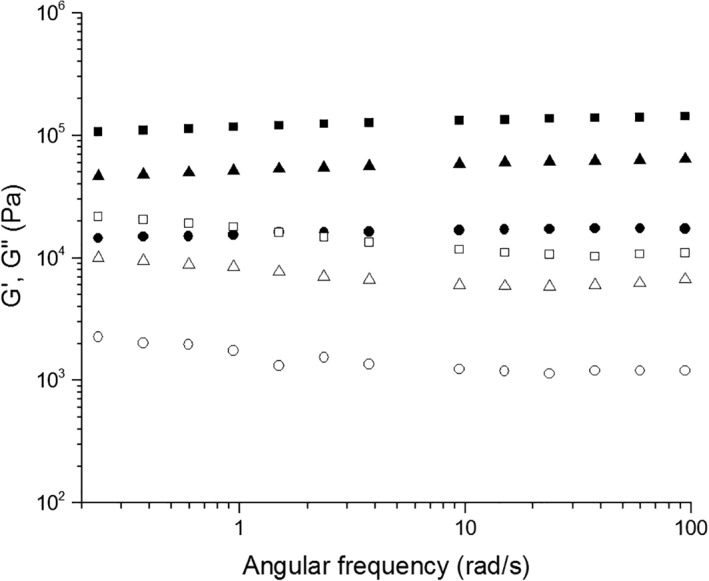
Dynamic frequency sweep at 0.2% of strain of H1 (●) H2 (▲) H3 (■) hydrogels; Closed symbols are the storage modulus (G′’) and open symbols are the loss modulus (G″).

In order to investigate the viscoelastic properties of the SA5N peptide based hydrogels, the elastic (G′) and loss (G″) moduli were measured by shear oscillatory rheology. Hydrogels H1‐H3, formed by SA5N peptide at different concentrations were analyzed. The data as a function of radial frequency are shown in Figure 4. A weak frequency dependence of G′ and G″ for all three samples is shown and G′ was found to be higher than G″, which is a typical behavior of soft solid‐like materials, constituted of entangled networks. The G′ range covers very high values, from 10 kPa for SA5N 0.7% to 120 kPa for 2% SA5N, as shown in Figure 4.

**Figure 5:**
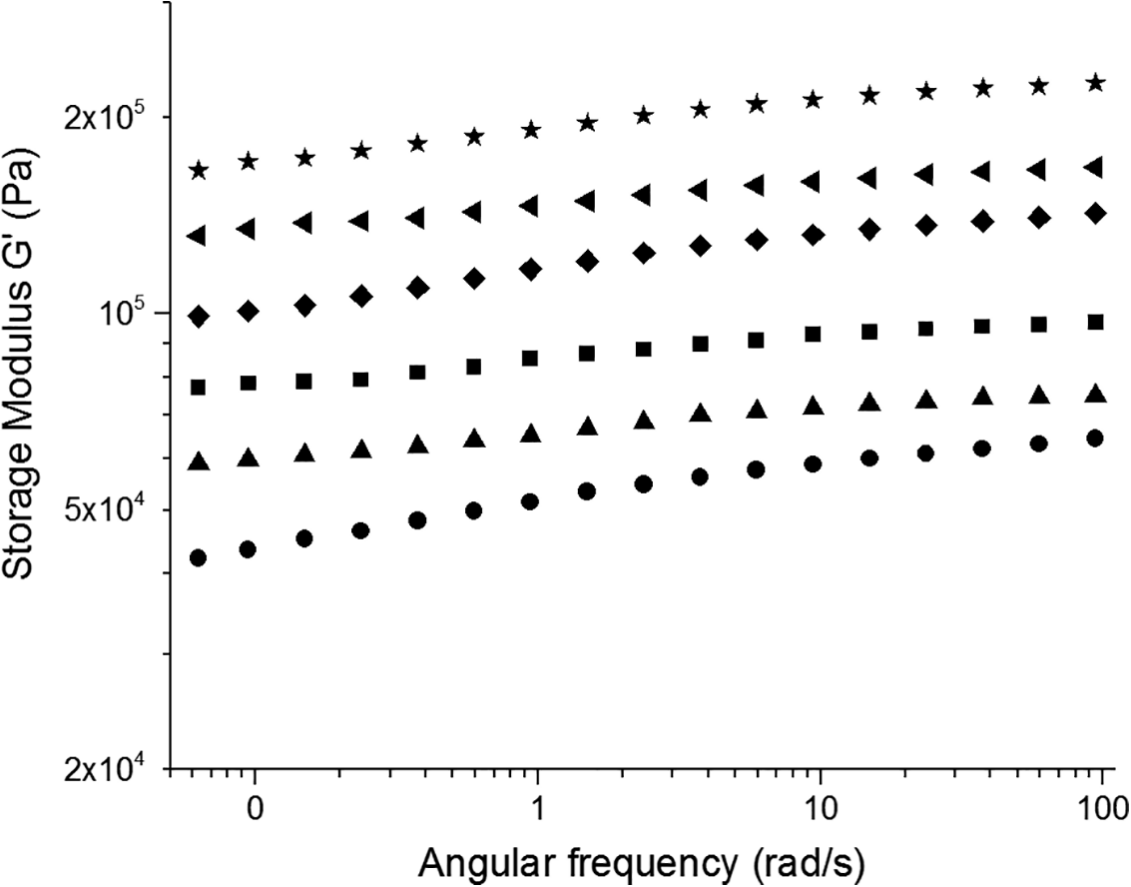
Dynamic frequency sweep at 0.2% strain of H2 (●); H4 (▲); H5 (■); H3 (♦); H6 (◄); H7 (★) hydrogels.

The rheological behavior of the SA5N hydrogels is modified by the addition of three‐block SA21 peptide (Fig. 5). The presence of SA21 induces a gradual increase of the G′ value with increasing amounts of SA21, observed for two different concentrations of SA5N (1% and 2%).

### 3.5 AFM microscopy

**Figure 6:**
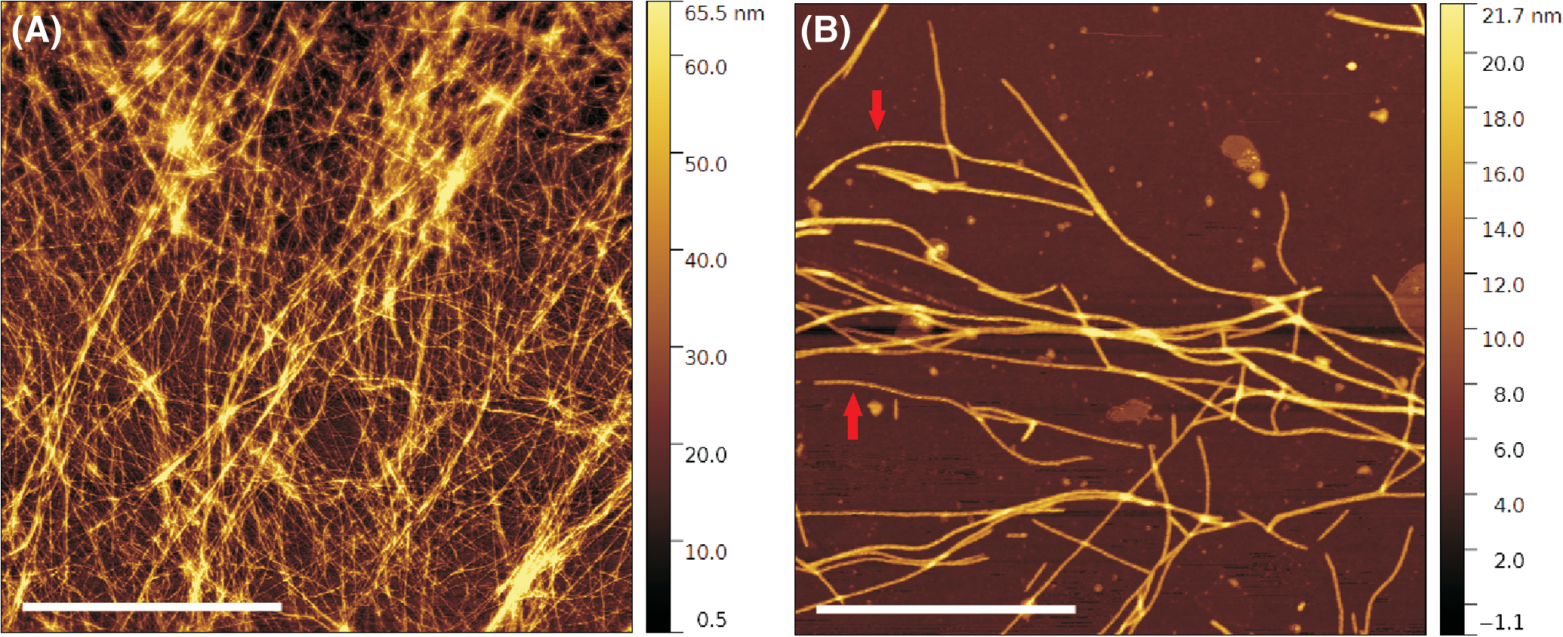
AFM topography images of H2 hydrogel. (A) Highly entangled network; (B) in a less dense region twisted–rope fibrils are discernible as indicated by the red arrows. Scale bars: 4 μm (a); 2 μm (b).

Atomic force microscopy was used to explore the nature of the network structure of the different hydrogels with the aim to find a relationship between mechanical properties and morphology of the fibers. The AFM images were acquired on samples obtained by deposition of the hydrogels after 24 h of gelification and analyzed with intermittent contact mode. A dense network of entangled thin fibrils is clearly observed for H2 hydrogel [Fig. 6(A)]. When a less densely packed region is observed at higher magnification, more defined fibrils are visible. Additionally, twisted‐rope structures can be observed [Fig. 6(B)]. The thin and straight SA5N fibrils are regularly twisted with a pitch of ca. 70 nm, as shown in Figure S1, giving rise to a highly entangled network in accordance with rheological data. A higher order aggregation of the fibril network is not observed, while the diameters of the fibers range from 25 to 40 nm.

**Figure 7:**
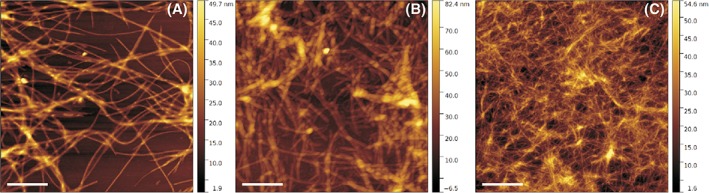
AFM topography images of H2 (A), H4 (B), and H5 (C) hydrogels. Scale bar: 1 μm.

Atomic force microscopy images of hydrogels with increasing amount of SA21 peptide are shown in Figure 7. The morphology of the SA5N hydrogels changes as a function of SA21 concentration. Higher quantities of SA21 peptides promote the formation of more fiber entanglements. Likewise, a higher number of junction points are discernible and the mesh size is smaller.

### 3.6 TEM analysis

**Figure 8:**
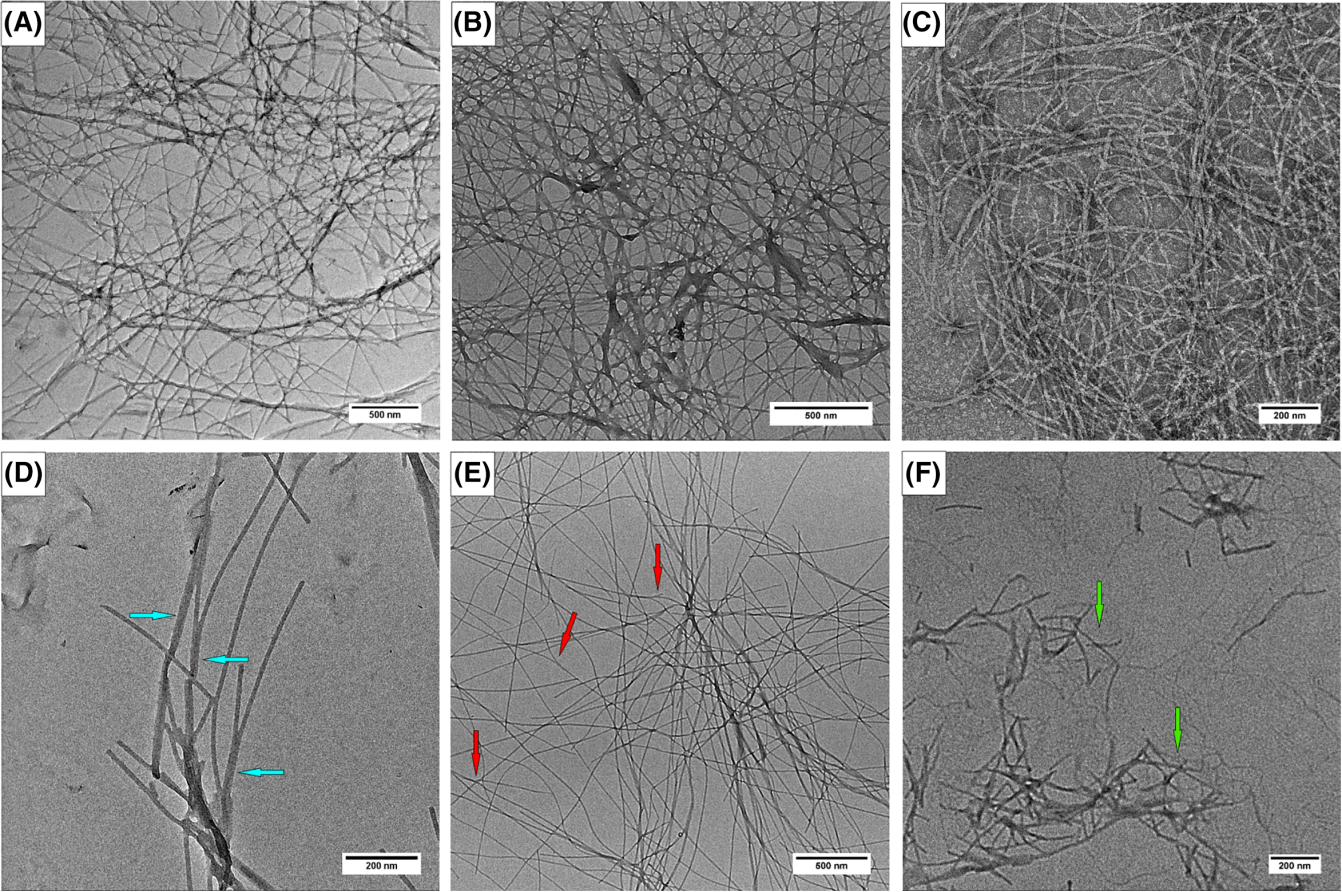
TEM images of H2 (A, D), H4 (B, E), H5 (C, F) hydrogels. Blue arrows in (D) indicate side‐by‐side aligned fibers in H2 hydrogel; red arrows in (E) indicate branching points in H4 hydrogel; green arrows indicate highly branched nanofibers observed in H5 hydrogel.

Transmission electron microscopy (TEM) was used to gain deeper insight into the nanoscale morphology of the hydrogels assemblies. Figure 8 shows TEM images of the tenfold diluted H2, H4, and H5 hydrogel samples. The presence of nanofibers, forming a densely entangled network is confirmed in all the samples. However, some differences are discernible in the less dense regions. H2 hydrogel is composed of straight rigid nanofibers with a mean diameter of 13.7 ± 1.5 nm. The nanofibers are often aligned side‐by‐side, as highlighted in Figure 8(D) by blue arrows. H4 hydrogel is composed of straight nanofibers of similar diameters, that show some sparsely distributed branching points [Fig. 8(E); red arrows]. Shorter and more flexible nanofibers are shown in the TEM images of H5 hydrogel, with widespread branching points [Fig. 8(F); green arrows]. While it is difficult to discriminate branching of individual fibrils from superposition of fibrils on top of each other in images showing crowded fibril structures [Fig. 8(A–C)],branching can be observed more clearly in the less densely packed regions imaged in Figure 8(D–F). These observations highlight the role of SA21 in changing the network topology of the hydrogels.

### 3.7 Cell culture

**Figure 9:**
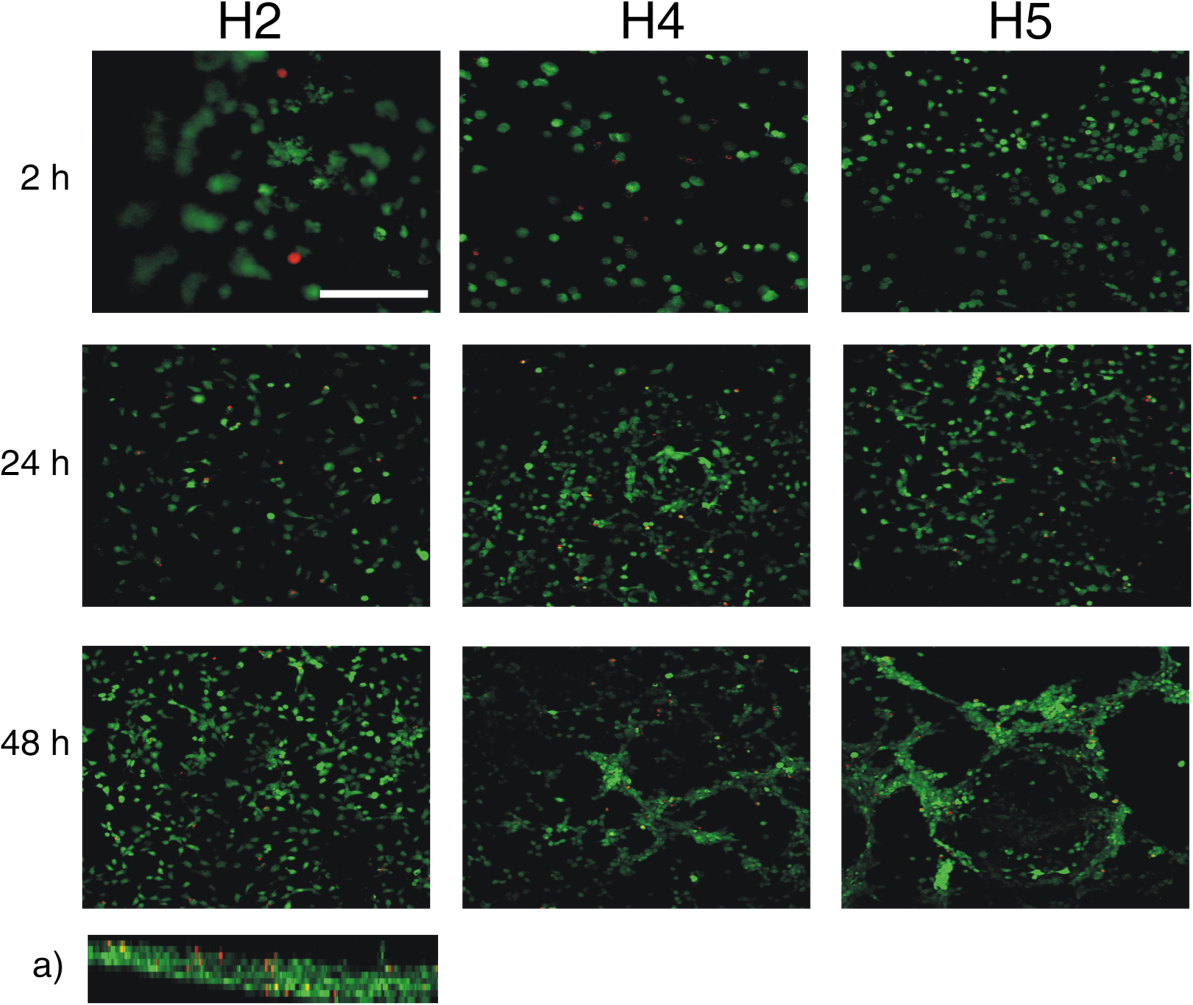
Confocal images (scale bar: 250 μm, magnification: 20×) obtained for 3 T3 fibroblast cultured on the surface H2, H4, and H5 hydrogels; axial image (*z*‐axis) (a) shows the multilayer cellular growth. Viable and dead cells are in green and red colors, respectively.

**Figure 10:**
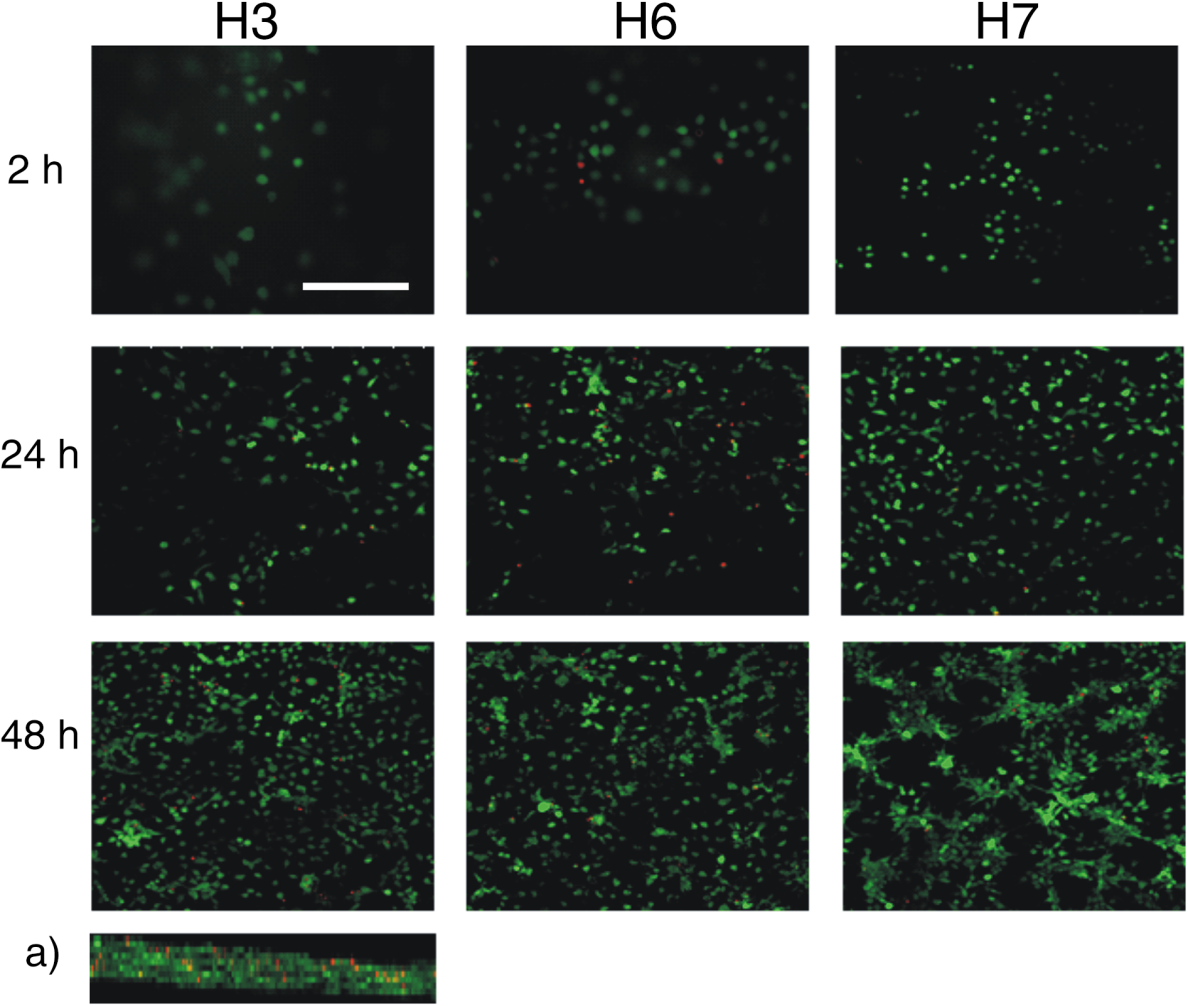
Confocal images (scale bar: 250 μm, magnification: 20×) obtained for 3 T3 fibroblast cultured on the surface of H3, H6, and H7 hydrogels showing viable (green) and dead cells (red); axial image (*z*‐axis) (a) shows the multilayer cellular growth.

The suitability of SA5N/SA21 hydrogels to support the culture of mammalian cells was investigated through the evaluation of murine fibroblasts (3T3 cells) viability when cultured on SA5N/SA21 gels. These cells are able to proliferate on scaffolds with a wide range of stiffness values, although they prefer more stiff matrices, where they undergo less apoptosis and increased proliferation.^17^, ^18^, ^48^, ^49^ Most of 3T3 cells were viable on SA5N/SA21 (H4, H5, H6, and H7) hydrogels over 48 h of culture (Figs. 9 and 10). Interestingly, 3T3 cells on such gels, tended to acquire a spread morphology and form cellular clusters and networks throughout the gels surface, which presumably followed the topography of the gels surface [Figs. 9(A) and 10(A)]. On the other hand, cells cultured on SA5N gels (H2 and H3 controls) remained dispersed throughout the gel surface without forming cell networks. Overall, cell viability imaging suggests that SA5N/SA21 gels support the viability of fibroblast cells and that the formation of cellular networks (cell–cell interactions) might be dependent on the presence of adhesion sites (elastin sequences) provided by SA5N/SA21 gels, since such cellular networks were not observed on H2 and H3 gels, even the latest, that offers higher stiffness than H4 and H5 gels.^17^, ^18^, ^48^, ^49^

**Figure 11:**
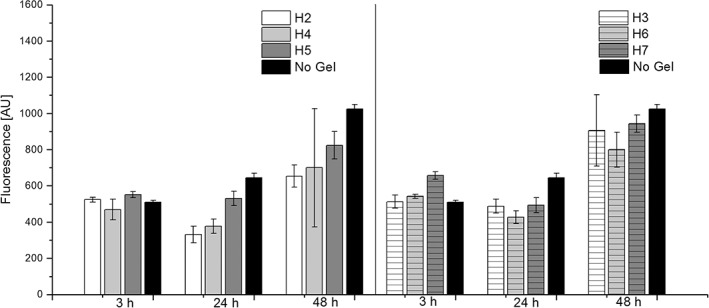
Metabolic activity measured by Alamar Blue assay of the 3 T3 cell culture on the peptide hydrogels. No Gel refers to cells grown on TCP. Data shows the mean of duplicate measurements. The error bars represent standard deviation.

To confirm the suitability of SA5N/SA21 gels to support cellular growth, the metabolic activity of fibroblasts on hydrogels was evaluated using Alamar Blue assay (AB). Cells cultured on cell culture plates (TCP) were used as a reference (Fig. 11). Cells cultured on different SA5N/SA21 gels (H2, H3, H4, H5, H6, and H7), showed a steady cell metabolic activity with no significant differences among samples over 48 h of culture, which nicely matches the observed confocal imaging.

After 3 h incubation metabolic activity was observed to be similar in all samples, which shows that there were no immediate cytotoxic effects of the gels on the cells. Metabolic activity measured at 24 h post‐seeding was reduced compared to 3 h incubation and the TCP control for all samples. This observation is commonly seen when cells are seeded on hydrogels.^6^ After 48 h of incubation the metabolic activity of cells grown on H2, H4, H5 hydrogels was slightly less than the cells grown on TCP. However, the metabolic activity of the cells grown on the stiffer hydrogels; H3, H6, and H7, was observed to be similar to those grown on the 2D plastic surface. Cells are known to respond to surface stiffness and 3T3 cells are no exception^17^, ^18^, ^48^, ^49^ These observations show that the gels did not appear to affect the metabolic activity of cells in the first 48 h after surface seeding.

## 4 DISCUSSION

Several peptides are able to form hydrogels and were explored as scaffolds in tissue engineering. Some of them were inspired by the yeast protein zuotin,^50^ other variants were *de novo* designed and are based on a ionic complementary design with alternating hydrophilic/hydrophobic sequences, which promote aggregation and gelation under appropriate conditions.^51^

In the present work, we were interested in β‐sheet forming peptides, inspired by amyloid‐like sequences.^52^ We have described the *de novo* design of a two‐peptide system, that allows to formulate hydrogels with tailored mechanical properties. This was achieved by designing a short peptide SA5N that self‐assembles and forms beta‐sheet fibers that above a critical gelation concentration forms a percolated networks and an hydrogel. The mechanical properties were controlled by the concentration of the peptide and the introduction of a second longer peptide designed with a central elastin‐inspired flexible sequence end‐capped at both ends with the self‐assembling peptide (SA21 peptide). The aim was to introduce physical cross‐links between beta‐sheet fibers to control the hydrogel mechanical properties. The occurrence in SA21 peptide of the elastin sequence (VPGVG)_2_ containing proline residues hinders the adoption of α‐helices as well as β‐sheet structures conferring high disorder to the backbone chain and introducing an elastic bridge between the SA5N fibers.^53^ As well known, elastin sequences are able to adopt highly flexible conformation populating mainly β‐turns, polyproline II (PPII) and unordered conformation, thus creating a high entropy state responsible for the elasticity of elastin.^54^, ^55^ Spectroscopic results by FT‐IR indicate that the self‐assembly of the peptides is based on the formation of antiparallel β‐sheets of SA5N peptide. Additionally, the presence of SA21 in the hydrogel does not interfere with the self‐assembly process, as the spectra are very similar for all the hydrogels tested suggesting that the SA5N corresponding sequences present in SA21 peptides are able to insert into the forming fibrils, while the central elastin sequence forms flexible structures predominantly populated by β‐turns and unordered structures.^33^

Rheological studies showed that G’ values observed for the SA5N hydrogels are dependent of peptide concentration and are an order of magnitude higher compared to those reported in the literature for other small peptides.^7^, ^26^, ^56^ The elastic storage moduli recorded for the H1, H2, H3 hydrogels are closer to those observed for Fmoc‐dipeptide based hydrogels, where the main driving forces for self‐assembly are based on an extensive π‐*π* stacking of the aromatic fluorenyl moiety.^57^, ^58^, ^59^ These data let us to infer that SA5N peptide hydrogels have a more complex network structure with higher numbers of fiber‐fiber interactions due to the presence of three aromatic phenyl groups in the side chains of the self‐assembling peptide. The aromatic rings of the Phe residues are positioned on both side of the peptide backbone retaining the possibility to interact by π–π interaction on different side of the formed fibers. This could contribute to the high G′ values of the hydrogels. Instead, in the case of ionic complementary peptides, the fibers present a hydrophilic and hydrophobic face and the aromatic groups are exposed only on one side.^22^ Rheological data of the two‐component peptide hydrogel shows a significant increasing of the stiffness, when SA21 peptide is added to the hydrogel. These data let us to propose that SA21 peptide participates in the network structure by insertion of the self‐assembling part of its sequence in the nanofibers and creating additional junction points. Supramolecular studies of the hydrogels performed by AFM and TEM have shown that the difference in mechanical stiffness of the hydrogels can be attributed to variances in the network topology of the hydrogels. While mono‐component hydrogels H1, H2, H3 are constituted by the same network topology at all concentrations but more densely packed; when introducing the SA21 peptide in the system a model with different network topology has to be proposed. As previously shown, SA21 alone is not able to form hydrogel networks, but forms nanospheres, through π–π interactions of the Phe residues.^33^ The AFM images of SA5N/SA21 two‐component peptide systems did not show any spherical structure, as SA21 alone did in the same solution condition (Fig. S8), suggesting that the mechanism of hydrogel formation was due to co‐assembly rather than self‐sorting of the mixtures, confirming an intimate insertion of SA21 in the nanofibers.

From TEM images we observe that the insertion of SA21 in the nanofiber promotes branching, probably responsible for changes the rheological behavior of the hydrogels. In particular, doping of the SA5N peptide hydrogel with small amounts of SA21 peptide (H4 hydrogel) promotes the formation of a small number of “Y” shaped branching points that, however, significantly increase the storage modulus. On increasing the amount of SA21 peptide, the presence of a higher number of branching points could determine the formation of shorter nanofibers. In this way, the strengthening effect due to branching is counterbalanced by the shortening and kinking of the nanofibers. Branching of nanofibers was observed in several self‐assembling peptide, like branched peptides,^60^ Amyloid‐based β‐sheet peptide nanofibers^61^, ^62^ and β‐hairpin peptides composed of alternating hydrophilic and hydrophobic amino acids.^56^ The presence of branching points were assigned in the latter case to imperfections in the bilayer self‐assembly, typical of these peptides that produced an increased hydrogel strength. Also in the two‐component peptide system described by Boothroyd et al.^20^ the insertion of the double length peptide containing a small –GG– dipeptide linker sequence induces concurrently branching and parallel fiber formation, making it difficult to distinguish the specific effect of the network topology changes on the hydrogels strength. In the case of the SA21 peptide, the presence of the flexible 11 residue‐long elastin sequence avoids the parallel fiber alignment and induces predominantly branching. Previous studies have shown that the elastin VPGVG pentapeptide repeat can contain a highly dynamic turn structures in equilibrium with more disordered structures, defining highly flexible structures as linker that could contribute to the mechanical properties of the hydrogel. This suggests that by appropriate peptide design it is possible to modulate the network topology and consequently the strength of the peptides hydrogel. Finally, the viability of fibroblast was evaluated for the hydrogels. Further studies on cell viability of different cell type as a function of hydrogel stiffness are under investigation.

## 5 CONCLUSIONS

The potential of the two‐component SAPH in tissue engineering is mainly due to the versatility of the system, as an appropriate sequence design can tune several properties, including stiffness and biological effects. In comparison to other self‐assembling peptides used in regenerative medicine, these peptides are extremely short, spontaneously self‐assemble in aqueous conditions, and form very strong scaffolds without chemical cross‐linkers.

The aggregation of the fibrils within the two‐component peptide system gives rise to a dense 3D network, which entraps high quantities of water and provides high and steady mechanical properties, favoring cellular growth. These kinds of scaffolds have shown a high biocompatibility and low cellular toxicity, supporting the attachment and proliferation of NIH 3T3 fibroblast cells. The inherent amino acid composition of SA5N/SA21 gels is advantageous in terms of biocompatibility, since the elastin sequence has allowed the growth of fibroblast cells. In order to use SA5N/SA21 gels for tissue engineering and regenerative medicine applications, the suitability of such gels to support different 3D culture cells has to be tested and is part of a forthcoming work.

## Supporting information


**Figure S1:** RP‐HPLC chromatograms of SA5N peptide after purification.
**Figure S2:** ^1^H‐NMR of SA5N peptide recorded in DMSO‐*d*_6_
**Figure S3:** ESI‐MS spectrum of SA5N peptide (*m/z* 633.40 [M + H]^+^, 633.303 calcd. For [C_33_H_41_N_6_O_7_]^+^)
**Figure S4:** RP‐HPLC chromatograms of SA21 peptide after purification.
**Figure S5:** ^1^H‐NMR of SA5N peptide recorded in DMSO‐*d*_6_
**Figure S6:** ESI‐MS spectrum of SA21 peptide (*m/z* 1063.52966 [M + 2H]^2+^, 1063.25108 calcd. For [C_106_H_143_N_21_O_26_]^2+^)
**Figure S7:** AFM topography image of H2 hydrogel (scale bar: 1 μm); height profile along the fiber 1; cross‐section of fiber 5 as indicated in the topography image.
**Figure S8:** AFM topography image of SA21 peptide deposited on Silicon wafer after dilution in 5% DMSO in water. (Scale bar = 2 μm)(top); 3D rendering of a magnified region (bottom).
