# Supplementary material for: Tuning of hydrogel stiffness using a two‐component peptide system for mammalian cell culture
Source: J Biomed Mater Res A. 2018 Nov 19;107(3):535–44. doi: 10.1002/jbm.a.36568 (PMC6587839; doi:10.1002/jbm.a.36568)
Supplement: Supplementary file 1 — Figure S1: RP‐HPLC chromatograms of SA5N peptide after purification. Figure S2: 1H‐NMR of SA5N peptide recorded in DMSO‐d 6 Figure S3: ESI‐MS spectrum of SA5N peptide (m/z 633.40 [M + H]+, 633.303 calcd. For [C33H41N6O7]+) Figure S4: RP‐HPLC chromatograms of SA21 peptide after purification. Figure S5: 1H‐NMR of SA5N peptide recorded in DMSO‐d 6 Figure S6: ESI‐MS spectrum of SA21 peptide (m/z 1063.52966 [M + 2H]2+, 1063.25108 calcd. For [C106H143N21O26]2+) Figure S7: AFM topography image of H2 hydrogel (scale bar: 1 μm); height profile along the fiber 1; cross‐section of fiber 5 as indicated in the topography image. Figure S8: AFM topography image of SA21 peptide deposited on Silicon wafer after dilution in 5% DMSO in water. (Scale bar = 2 μm)(top); 3D rendering of a magnified region (bottom). [file JBM-107-535-s001.docx]

Supplementary Information:

**TUNING OF HYDROGEL STIFFNESS USING A TWO-COMPONENT PEPTIDE SYSTEM FOR MAMMALIAN CELL CULTURE**

Alessandra Scelsi^a,b^, Brigida Bochicchio^a^, Andrew Smith^c^, Victoria L.Workman^c^, Luis A. Castillo Diaz^,c,d^, Alberto Saiani^c^, Antonietta Pepe^a*^

^a^ Laboratory of Bioinspired Materials, Department of Science, University of Basilicata, Potenza, Italy.

^b^PhD School of Science, University of Basilicata, Potenza, Italy.

^c^School of Materials and Manchester Institute of Biotechnology, The University of Manchester, , Manchester, U.K

^d^Biotecnología Médica y Farmacéutica. Centro de Investigación y Asistencia en Tecnología y Diseño del Estado de Jalisco (CIATEJ), México.


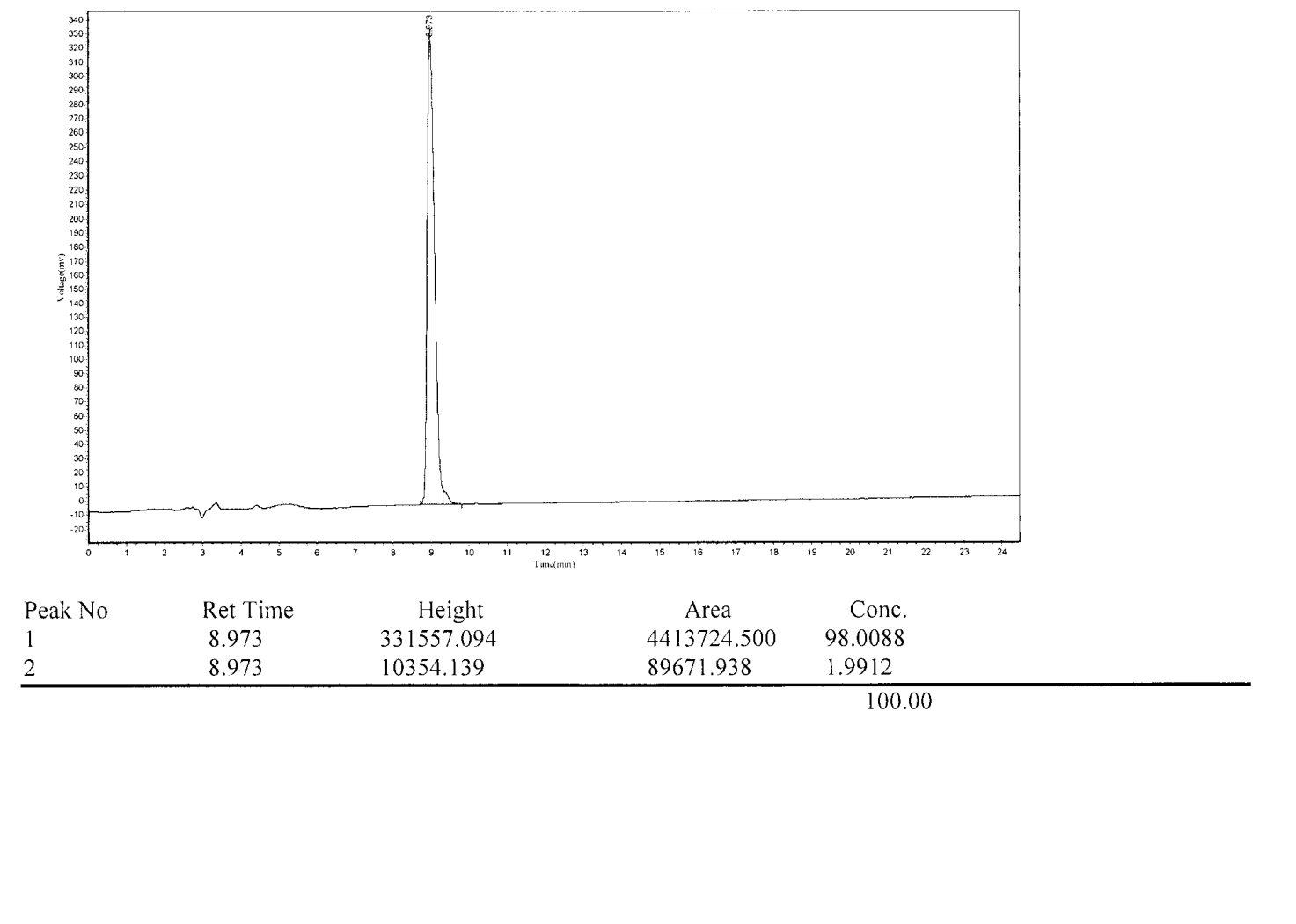
Figure S1: RP-HPLC chromatograms of SA5N peptide after purification.


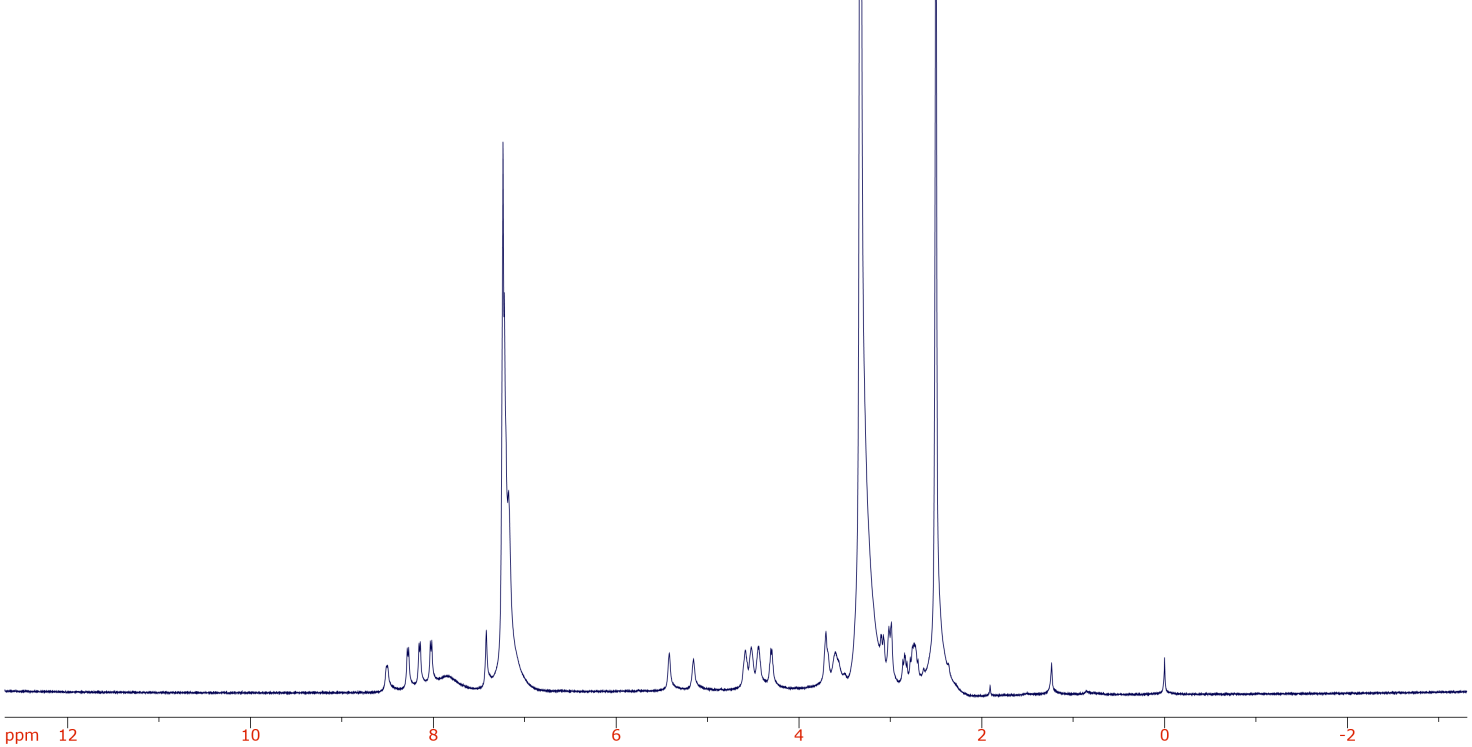
 Figure S2: ^1^H-NMR of SA5N peptide recorded in DMSO-*d_6_*





Figure S3: ESI-MS spectrum of SA5N peptide. (m/z 633.40 [M+H]^+^, 633.303 calcd. for [C_33_H_41_N_6_O_7_]^+^)


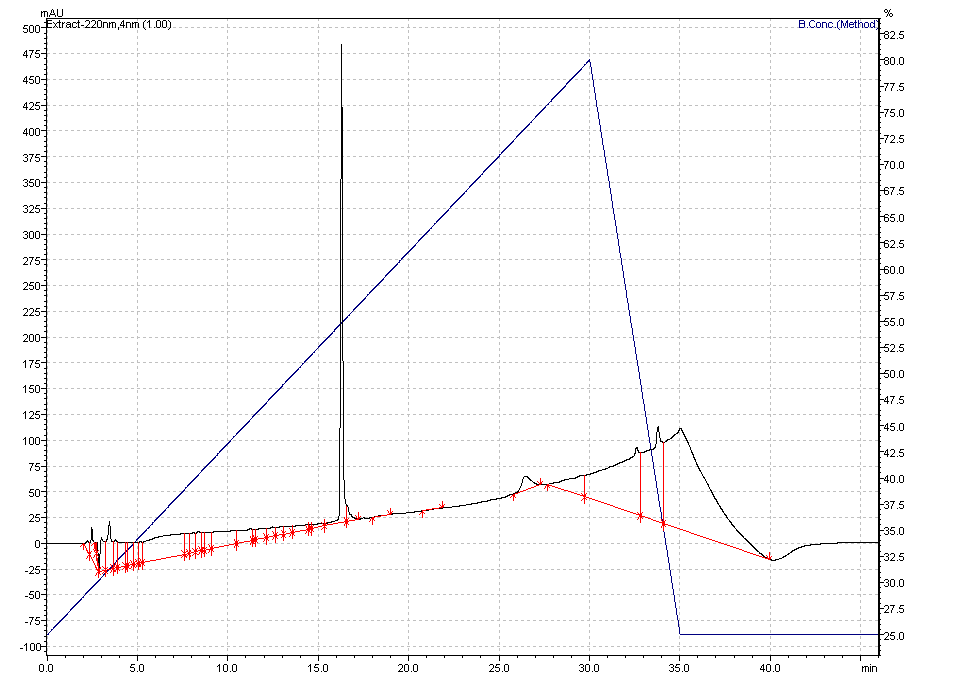
Figure S4: RP-HPLC chromatograms of SA21 peptide after purification.


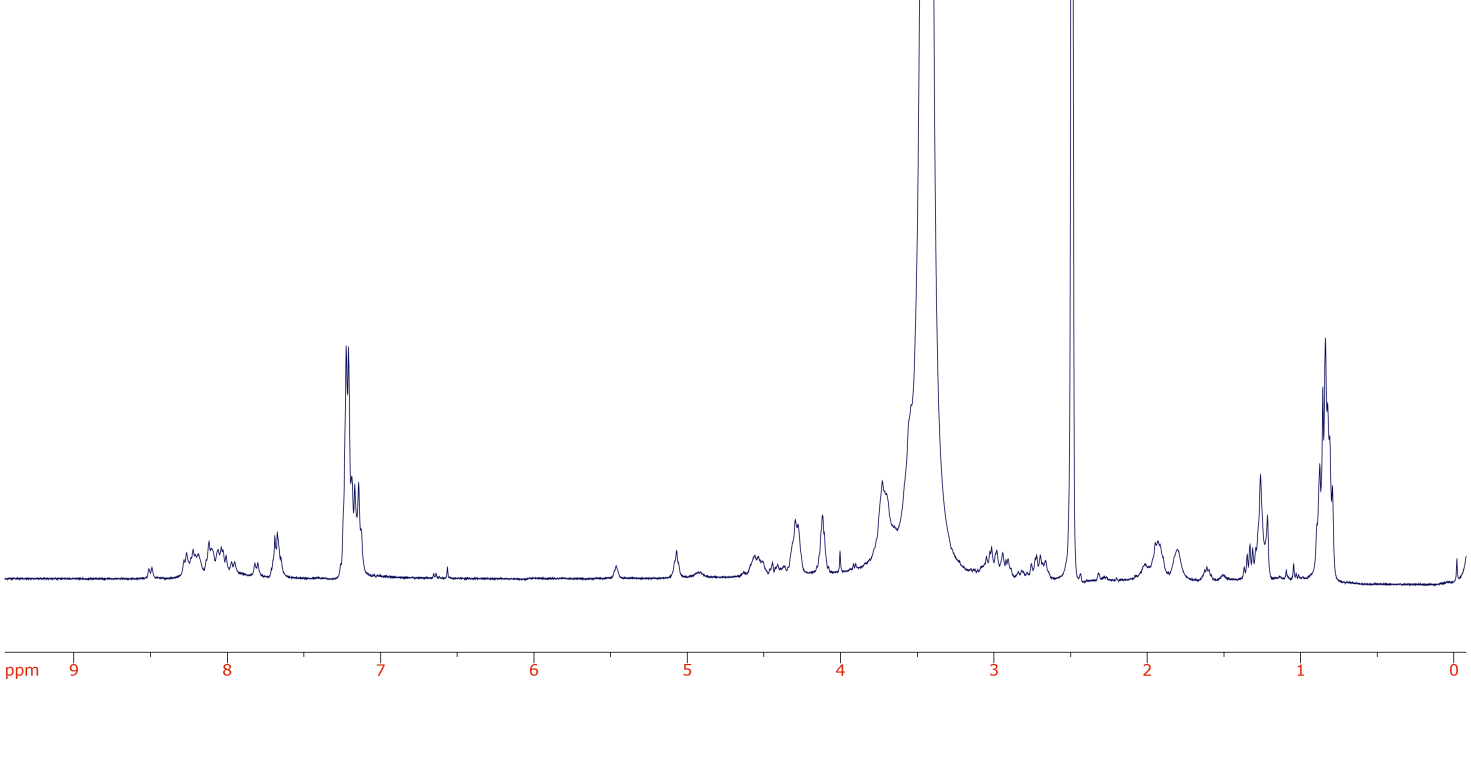
Figure S5: ^1^H-NMR of SA5N peptide recorded in DMSO-*d_6_*

Figure S6: ESI-MS spectrum of SA21 peptide. (m/z 1063.52966 [M+2H]^2+^, 1063.25108 calcd. for [C_106_H_143_N_21_O_26_]^2+^)




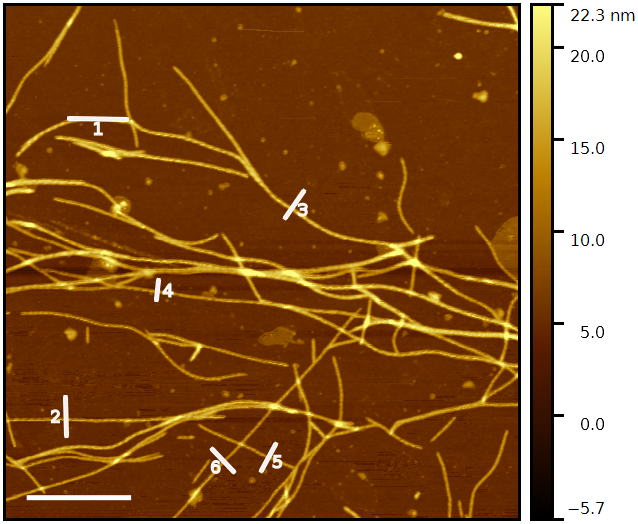




Figure S7: AFM topography image of H2 hydrogel (scale bar: 1 μm) ; height profile along the fiber 1; cross-section of fiber 5 as indicated in the topography image.


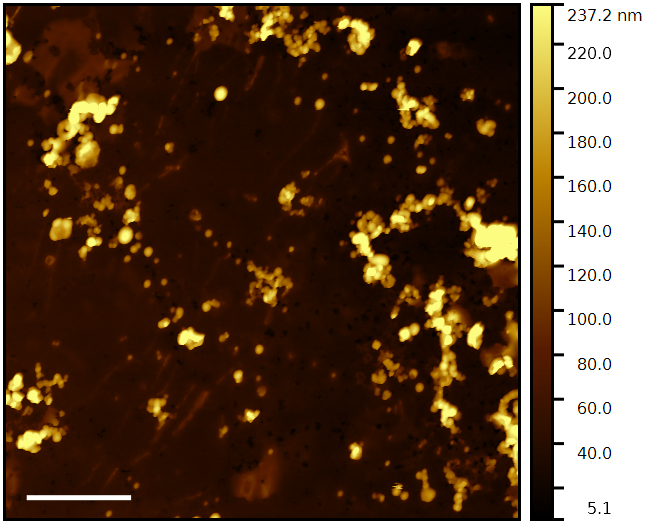


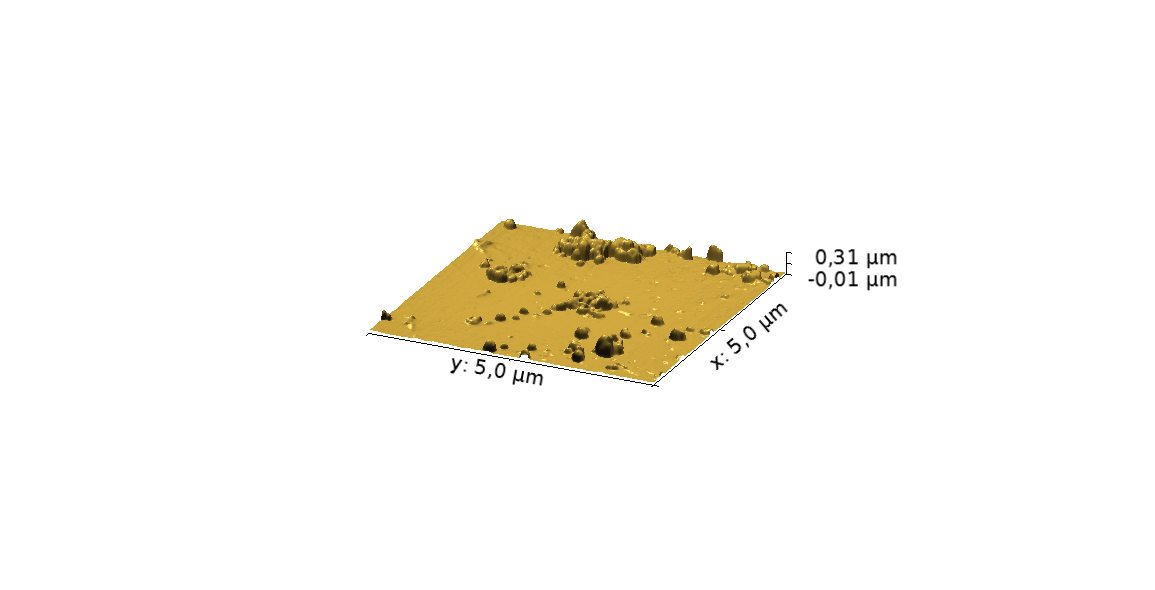


Figure S8: AFM topography image of SA21 peptide deposited on Silicon wafer after dilution in 5% DMSO in water. (Scale bar= 2 μm)( top); 3D rendering of a magnified region (bottom).
